# A new supra­molecular cobalt(II) complex based on 1,10-phenanthroline and 4-nitro­phthalate ligands

**DOI:** 10.1107/S2056989026006419

**Published:** 2026-06-26

**Authors:** Naima Karimova, Maftuna Primkulova, Lidiya Izotova, Gulvar Muqumova, Jabbor Suyunov, Nurmuhammad Boltayev

**Affiliations:** a Termez State University, A Navoiy Str, 43, Termez, 190100, Uzbekistan; b Denau Institute of Entrepreneurship and Pedagogy, Bog Str, 112, Denau, 733500, Uzbekistan; c Institute of Bioorganic Chemisty, UzAS, M.Ulugbek Str. 83, 100125, Tashkent, Uzbekistan; Institute of Chemistry, Chinese Academy of Sciences

**Keywords:** crystal structure, binuclear cobalt(II) complex, 1,10-phenanthroline, 4-nitro­phthalate

## Abstract

The title compound, bis­(μ-2-carb­oxy-4-nitro­benzoato-κ^2^*O*^1^:*O*^1′^)bis­[bis­(1,10-phenanthroline-κ^2^*N*,*N*′)cobalt(II)] bis­(2-carb­oxy-4-nitro­benzoate) tetra­hydrate, [Co_2_(C_8_H_4_NO_6_)_2_(C_12_H_8_N_2_)_4_](C_8_H_4_NO_6_)_2_·4H_2_O, comprises a centrosymmetric dinuclear cobalt(II) complex dication, two hydrogen 4-nitro­phthalate anions and four water mol­ecules of crystallization. The two Co^II^ atoms are linked by two μ-hydrogen 4-nitro­phthalato ligands, generating a centrosymmetric dinuclear unit. Each cobalt(II) centre adopts a distorted octa­hedral coordination geometry defined by four N atoms from two chelating 1,10-phenanthroline ligands and two O atoms from two symmetry-related bridging hydrogen 4-nitro­phthalate ligands. In the crystal, O—H⋯O and C—H⋯O hydrogen bonds link the ionic components into a three-dimensional supra­molecular framework, which is further reinforced by aromatic π–π stacking inter­actions between neighbouring phenanthroline and hydrogen 4-nitro­phthalate rings, with centroid-to-centroid separations ranging from 3.501 (5) to 3.687 (4) Å. Hirshfeld surface analysis shows that O⋯H/H⋯O contacts make the largest contribution (38.1%) to the crystal packing, confirming the dominant role of hydrogen bonding in consolidating the crystal structure.

## Abstract 2

A binuclear cobalt(II) complex assembled from 1,10-phenanthroline and 4-nitro­phthalate ligands features carboxyl­ate-bridged metal centres in distorted octa­hedral coordination environments. The crystal packing is further consolidated by O—H⋯O hydrogen bonds and aromatic π–π stacking inter­actions, resulting in a three-dimensional supra­molecular network.

## 1. Chemical context

Mixed-ligand cobalt(II) complexes containing aromatic N-donor and polycarboxyl­ate ligands continue to attract attention because of their structural diversity and supra­molecular assembly patterns (Sammes & Yahioglu, 1994 ▸; Bencini & Lippolis, 2010 ▸). In particular, 1,10-phenanthroline commonly forms stable chelating coordination environments, whereas nitro­phthalate ligands exhibit versatile coordination modes and hydrogen-bonding capabilities. As part of our ongoing studies of cobalt(II) complexes containing mixed N- and O-donor ligands, the title dinuclear complex incorporating 1,10-phenanthroline and hydrogen 4-nitro­phthalate ligands was synthesized and characterized by single-crystal X-ray diffraction analysis.
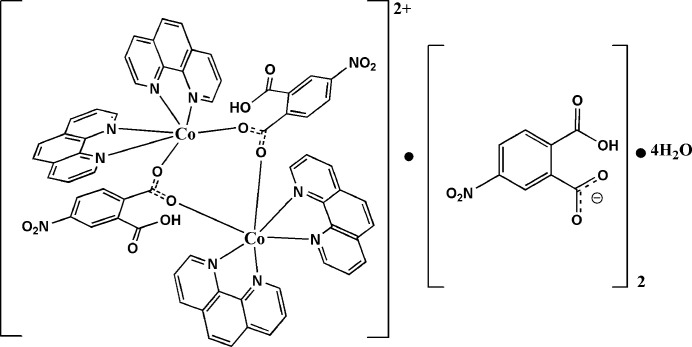


## 2. Structural commentary

**Figure 1:**
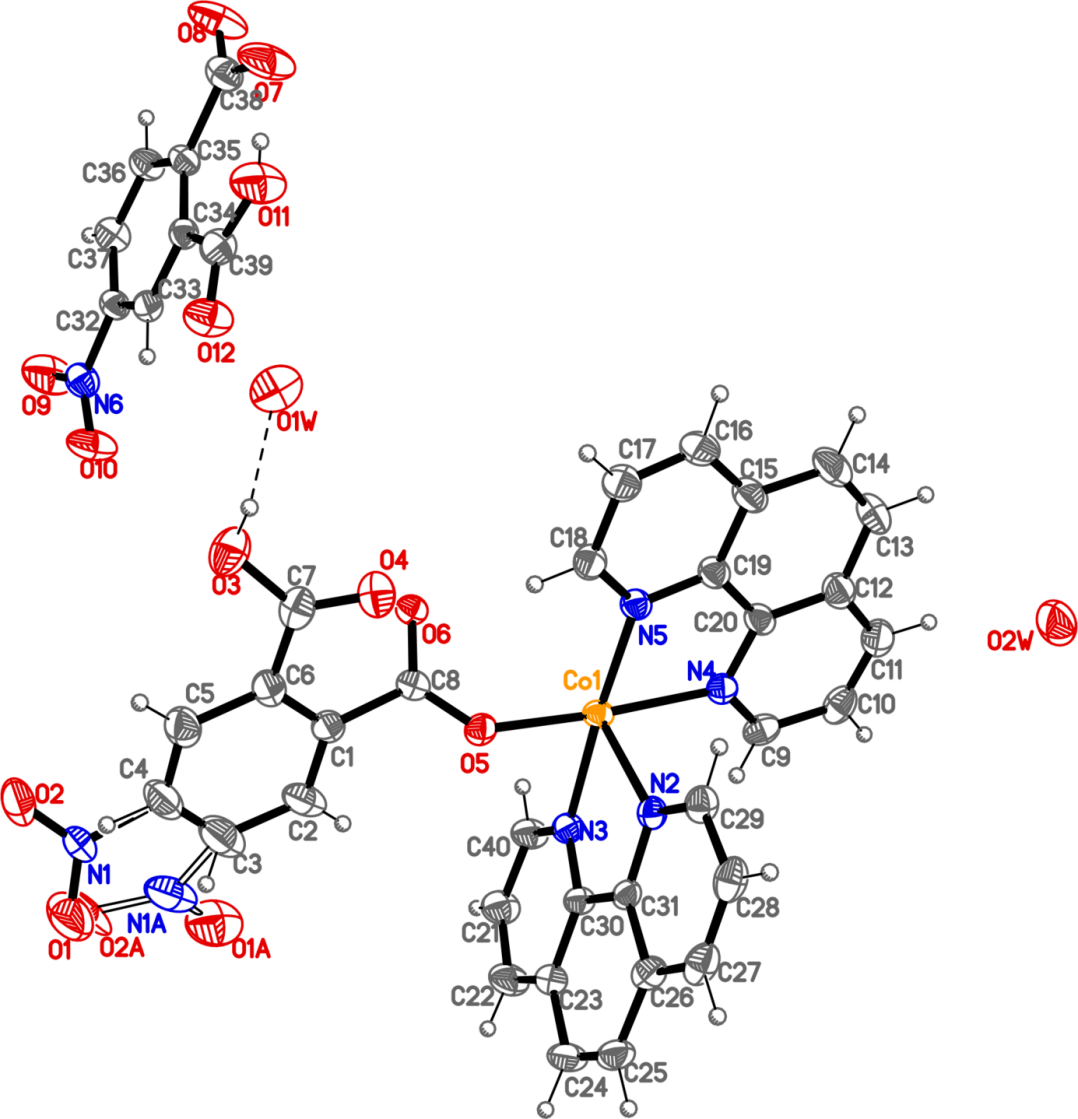
The asymmetric unit of the title compound showing the atom-labelling scheme. Displacement ellipsoids are drawn at the 30% probability level.

The mol­ecular structure of the title compound is shown in Fig. 1 ▸. The complex crystallizes in the triclinic space group *P*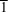. The asymmetric unit comprises one Co^II^ atom, two chelating 1,10-phenanthroline ligands, one μ_2_-bridging hydrogen 4-nitro­phthalate ligand, one uncoordinated hydrogen 4-nitro­phthalate anion and two solvent oxygen atoms (O1*W* and O2*W*) corresponding to highly disordered water mol­ecules. The complete centrosymmetric dinuclear complex dication is generated by inversion symmetry.

Each Co^II^ centre adopts a distorted octa­hedral CoN_4_O_2_ coordination geometry defined by four nitro­gen atoms from two chelating 1,10-phenanthroline ligands and two oxygen atoms from two symmetry-related hydrogen 4-nitro­phthalate ligands. The Co—O bond length is 2.070 (4) Å, while the Co—N bond distances range from 2.126 (4) to 2.169 (4) Å (Table 1 ▸). The *cis* angle O5—Co1—O6^i^ is 91.94 (13)°, while the *trans* angles N3—Co1—N5 and O5—Co1—N4 are 173.52 (16) and 171.16 (16)°, respectively, indicating only a slight distortion from an ideal octa­hedral geometry.

The coordinated hydrogen 4-nitro­phthalate ligand adopts a μ_2_-κ*O*:κ*O*′ bridging coordination mode, linking two symmetry-related cobalt(II) centres into a centrosymmetric dinuclear complex dication with an intra­molecular Co⋯Co separation of 4.795 (2) Å. Within the coordinated carboxyl­ate group, the C—O bond distances [1.254 (6) and 1.273 (6) Å] are consistent with electron delocalization, whereas the uncoordinated carb­oxy­lic group exhibits unequal C—O bond lengths [1.237 (7) and 1.326 (7) Å], confirming its protonated nature.

The coordinated 1,10-phenanthroline ligands are essentially planar and provide extended aromatic surfaces that participate in significant inter­molecular π–π stacking inter­actions, which, together with the hydrogen-bonding network, contribute to the cohesion of the crystal packing and the formation of a three-dimensional supra­molecular architecture.

## 3. Supra­molecular features

**Figure 2:**
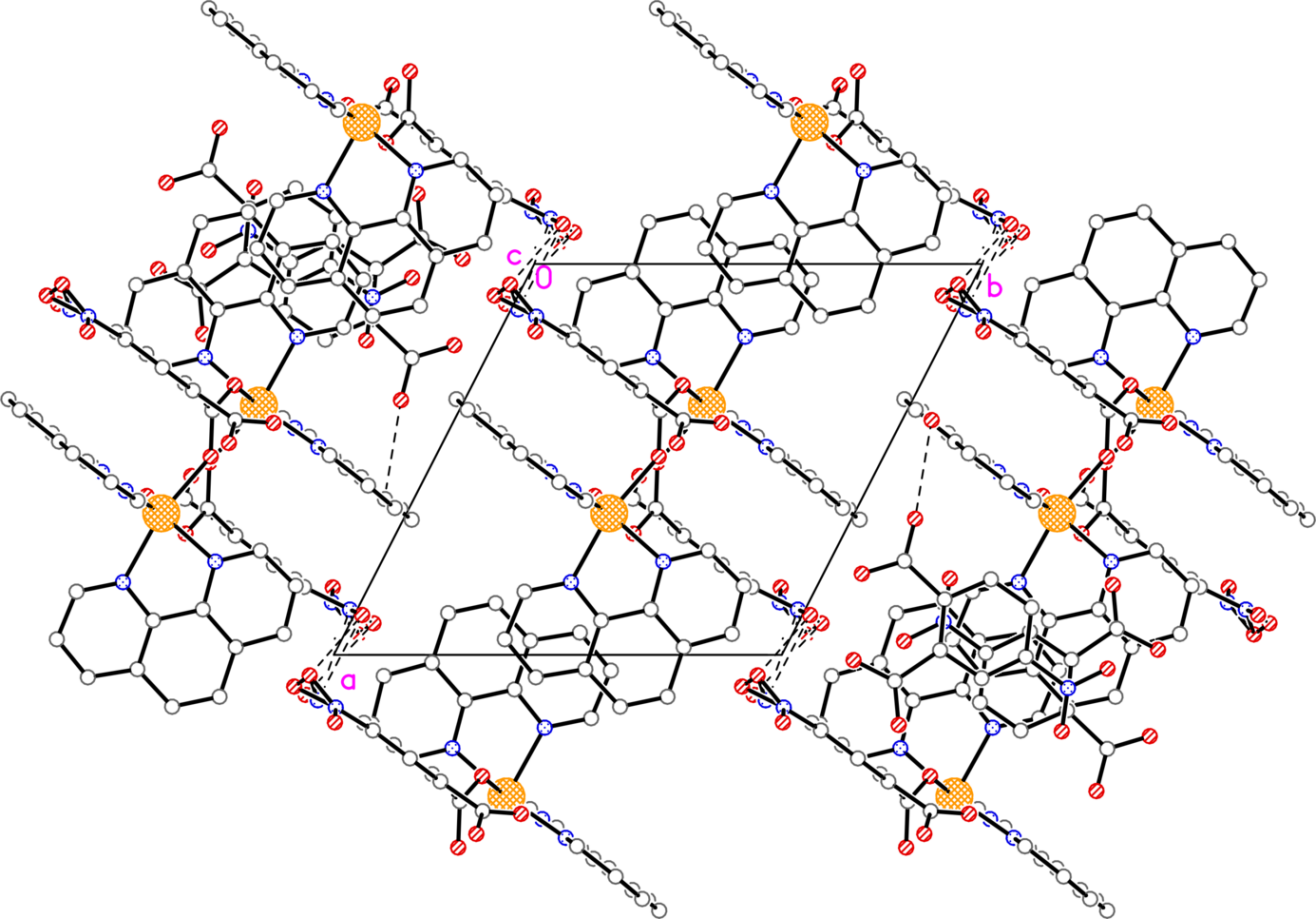
Crystal packing of the title compound viewed along the [001] direction.

The crystal packing is governed by a combination of classical O—H⋯O and weak C—H⋯O hydrogen bonds (Table 2 ▸), together with significant aromatic π–π stacking inter­actions (Fig. 2 ▸). The uncoordinated hydrogen 4-nitro­phthalate anions act as both hydrogen-bond donors and acceptors, whereas the solvent water oxygen atoms serve as hydrogen-bond acceptors. Collectively, these inter­actions link the centrosymmetric dinuclear complex dications, hydrogen 4-nitro­phthalate anions and solvent species into a three-dimensional supra­molecular architecture.

Several significant π–π stacking inter­actions are observed between the aromatic rings of the coordinated 1,10-phenanthroline ligands and the hydrogen 4-nitro­phthalate ligands. The shortest inter­action occurs between the pyridine ring of one 1,10-phenanthroline ligand (N4/C9–C12/C20; *Cg*5) and the benzene ring of a hydrogen 4-nitro­phthalate ligand (C1–C6; *Cg*7), with a centroid-to-centroid distance of 3.500 (5) Å, an inter­planar angle of 0.6° and a slippage of 1.03 Å, indicating an almost ideal face-to-face arrangement. Additional significant contacts are observed: *Cg*3⋯*Cg*3^i^ [3.600 (4) Å, 0.0°, 1.19 Å], *Cg*7⋯*Cg*8^i^ [3.609 (5) Å, 1.4°, 1.42 Å] and *Cg*9⋯*Cg*10^i^ [3.687 (4) Å, 2.7°, 1.18 Å] [*Cg*3, *Cg*7, *Cg*8, *Cg*9 and *Cg*10 are the centroids of the N2/C26–C29/C31, C1–C6, C12–C15/C19/C20, C23–C26/C30/C31 and C32–C37 rings, respectively; symmetry code: (i) −*x* + 2, −*y* + 1, −*z* + 1]. These geometrical parameters indicate efficient overlap of the aromatic π systems and contribute significantly to the cohesion of the crystal packing.

## 4. Hirshfeld surface analysis

**Figure 3:**
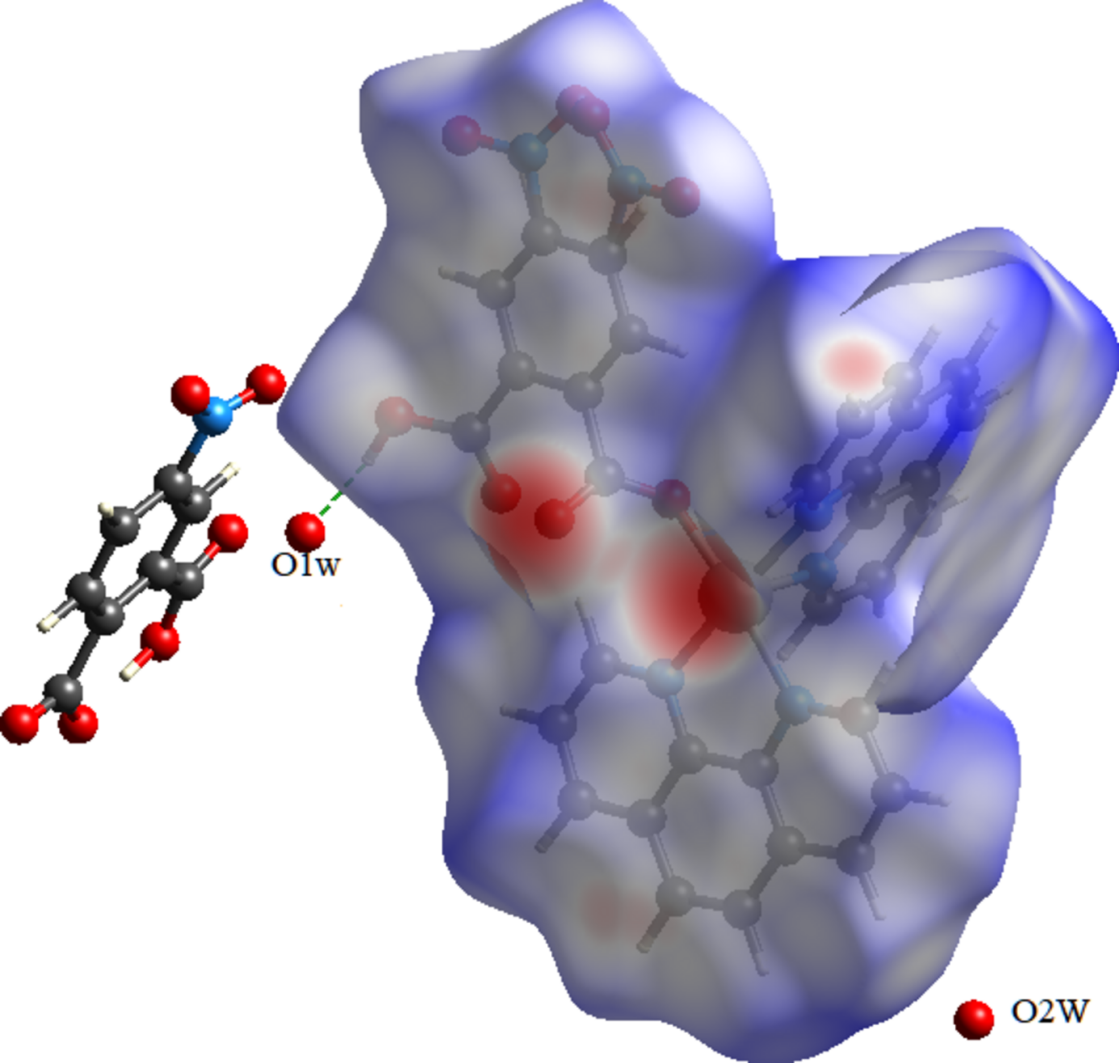
Hirshfeld surface mapped over *d*_norm_ showing short inter­molecular O⋯H/H⋯O contacts as red regions.

Hirshfeld surface analysis and the corresponding two-dimensional fingerprint plots were generated using *CrystalExplorer21.5* (Spackman *et al.*, 2021 ▸) to investigate the inter­molecular inter­actions responsible for the crystal packing. The Hirshfeld surface mapped over *d*_norm_ and the associated fingerprint plots are shown in Figs. 3 ▸ and 4 ▸, respectively.

The O⋯H/H⋯O contacts make the largest contribution to the Hirshfeld surface (38.1%), confirming that hydrogen bonding involving the carboxyl­ate, carb­oxy­lic acid and nitro oxygen atoms plays the dominant role in consolidating the crystal structure. H⋯H contacts account for 21.7% of the surface, reflecting the contribution of van der Waals inter­actions, whereas C⋯H/H⋯C contacts contribute 13.9%, indicating numerous weak inter­molecular C⋯H contacts within the crystal packing. Smaller contributions arise from O⋯C/C⋯O (6.1%), O⋯O (4.2%) and O⋯N/N⋯O (1.2%) contacts.

The Hirshfeld surface analysis is consistent with the crystallographic study, demonstrating that the crystal packing is governed primarily by classical hydrogen bonding, supplemented by weak inter­molecular contacts and significant aromatic π–π stacking inter­actions, which together generate the observed three-dimensional supra­molecular architecture.

## 5. Database survey

A search of the Cambridge Structural Database (CSD, Version 2025.3.1, update of February 2026; Groom *et al.*, 2016 ▸) revealed several cobalt(II) complexes containing the 4-nitro­phthalate ligand. Representative examples include HOJHOF (Li *et al.*, 2014 ▸), JUYREC and JUYRIG (Wang *et al.*, 2015*a* ▸), and LUDJUR (Yin & Li, 2015 ▸), in which the 4-nitro­phthalate ligand adopts various bridging coordination modes and gives rise to one-dimensional or higher-dimensional coordination architectures.

A separate search for cobalt(II) complexes containing both 1,10-phenanthroline and aromatic polycarboxyl­ate ligands identified several structurally related dinuclear complexes, including AJIYID (Wang *et al.*, 2015*b* ▸) and HUBCOY and HUBCUE (Wang *et al.*, 2015*c* ▸). In these compounds, the Co^II^ centres exhibit distorted octa­hedral coordination geometries defined by nitro­gen atoms from chelating 1,10-phenanthroline ligands and oxygen atoms from bridging carboxyl­ate groups.

The title compound displays structural features characteristic of both families, combining a centrosymmetric dinuclear cobalt(II) core bridged by hydrogen 4-nitro­phthalate ligands with chelating 1,10-phenanthroline ligands. A search of the current version of the CSD revealed no previously reported cobalt(II) complex containing both 1,10-phenanthroline and 4-nitro­phthalate ligands. To the best of our knowledge, the present structure therefore represents the first crystallographically characterized example of this type.

## 6. Synthesis and crystallization

The title compound was synthesized from cobalt(II) chloride hexa­hydrate, 4-nitro­phthalic acid and 1,10-phenanthroline using a molar ratio of 1:1:0.5. 4-Nitro­phthalic acid (1.00 mmol, 0.211 g) was dissolved in *N,N*-di­methyl­formamide (DMF), 1,10-phenanthroline (0.50 mmol, 0.090 g) in ethanol, and cobalt(II) chloride hexa­hydrate (1.00 mmol, 0.238 g) in distilled water. The solutions of 4-nitro­phthalic acid and cobalt(II) chloride hexa­hydrate were mixed and stirred magnetically for 20 min, after which the 1,10-phenanthroline solution was added dropwise. The resulting reaction mixture was stirred at 333 ± 0.5 K for a further 20 min. The clear solution was then left to stand at room temperature in a loosely covered vessel at pH ≃ 6.0. After 12 days, bright-red prismatic crystals suitable for single-crystal X-ray diffraction analysis were obtained. The crystals were collected by filtration and dried in air.

## 7. Refinement

Crystal data, data collection and structure refinement details are summarized in Table 3 ▸. Hydrogen atoms bonded to carbon atoms were placed in calculated positions and refined using a riding model, with C—H = 0.93 Å and *U*_iso_(H) = 1.2*U*_eq_(C). The hydrogen atoms of the carb­oxy­lic acid groups were located in difference-Fourier maps and subsequently refined using a riding model with O—H = 0.82 Å and *U*_iso_(H) = 1.5*U*_eq_(O). The nitro group of the coordinated hydrogen 4-nitro­phthalate ligand is disordered over two orientations and was refined using a split-atom model with refined site-occupancy factors of 0.51 (1) and 0.49 (1). Similarity restraints were applied to the N—O bond distances and anisotropic displacement parameters of the disordered atoms. Two solvent oxygen atoms (O1*W* and O2*W*), assigned to water mol­ecules of crystallization, were located in difference-Fourier maps and refined anisotropically. The corresponding hydrogen atoms could not be identified reliably in difference-Fourier maps and were therefore not included in the refinement.

## Supplementary Material

Crystal structure: contains datablock(s) I. DOI: 10.1107/S2056989026006419/nx2037sup1.cif

Structure factors: contains datablock(s) I. DOI: 10.1107/S2056989026006419/nx2037Isup3.hkl

CCDC reference: 2563257

Additional supporting information:  crystallographic information; 3D view; checkCIF report

## Figures and Tables

**Figure 4:**
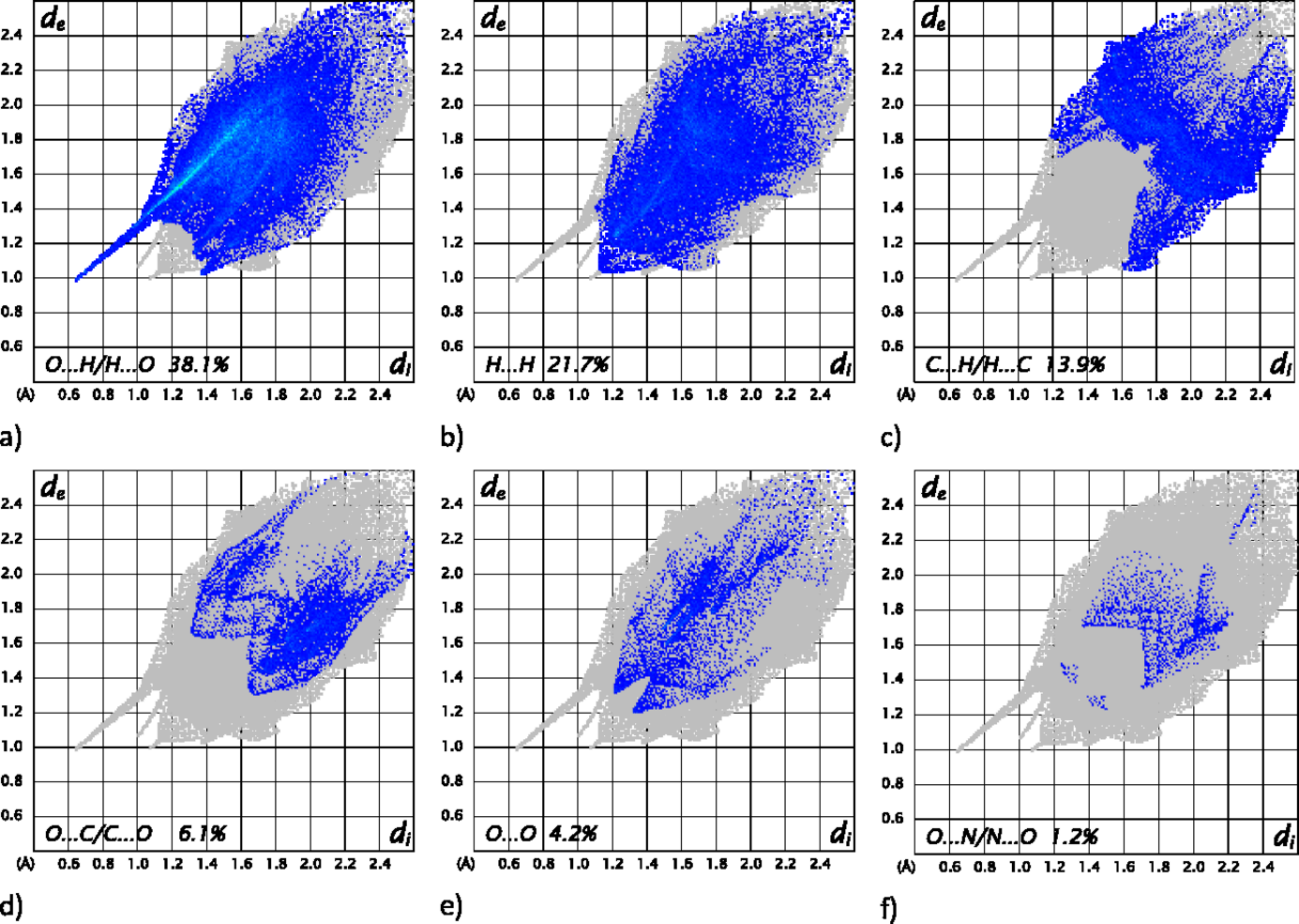
Two-dimensional fingerprint plots showing the percentage contributions of (*a*) O⋯H/H⋯O, (*b*) H⋯H, (*c*) C⋯H/H⋯C, (*d*) O⋯C/C⋯O, (*e*) O⋯O and (*f*) O⋯N/N⋯O contacts.

**Table 1:** Selected geometric parameters (Å, °)

Co1—O5	2.070 (4)	O6—C8	1.273 (6)
Co1—N5	2.126 (4)	O5—C8	1.254 (6)
Co1—N3	2.126 (4)	O4—C7	1.237 (7)
Co1—N4	2.137 (5)	O3—C7	1.326 (7)
Co1—N2	2.169 (4)		
			
O5—Co1—O6^i^	91.94 (13)	O6^i^—Co1—N2	166.84 (15)
N5—Co1—N3	173.52 (16)	N3—Co1—N2	77.25 (16)
O5—Co1—N4	171.16 (16)	O5—C8—O6	124.9 (5)
N5—Co1—N4	77.74 (18)	O4—C7—O3	122.5 (7)

Symmetry code: (i) 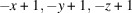.

**Table 2:** Hydrogen-bond geometry (Å, °)

*D*—H⋯*A*	*D*—H	H⋯*A*	*D*⋯*A*	*D*—H⋯*A*
O3—H3⋯O1*W*	0.82	1.78	2.549 (8)	155
O11—H11*A*⋯O7	0.82	1.58	2.400 (8)	178
C18—H18⋯O4	0.93	2.50	3.397 (8)	162
C9—H9⋯O12^i^	0.93	2.58	3.462 (8)	158
C40—H40⋯O6^i^	0.93	2.54	3.079 (6)	117
C21—H21⋯O7^ii^	0.93	2.42	3.329 (8)	165
C4—H4^b⋯O2A^b	0.93	2.00	2.49 (2)	112

Symmetry codes: (i) 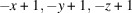; (ii) 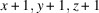.

**Table 3:** Experimental details

Crystal data	
Chemical formula	[Co_2_(C_8_H_4_NO_6_)_2_(C_12_H_8_N_2_)_4_](C_8_H_4_NO_6_)_2_·4H_2_O
*M*_r_	1743.16
Crystal system, space group	Triclinic, *P* 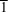
Temperature (K)	273
*a*, *b*, *c* (Å)	12.0908 (9), 13.2379 (15), 14.0783 (14)
α, β, γ (°)	111.315 (10), 92.136 (7), 114.312 (9)
*V* (Å^3^)	1866.1 (4)
*Z*	1
Radiation type	Cu *K*α
μ (mm^−1^)	4.32
Crystal size (mm)	0.2 × 0.1 × 0.05

Data collection	
Diffractometer	XCalibur
Absorption correction	Multi-scan (*CrysAlis PRO*; Agilent, 2014 ▸)
*T*_min_, *T*_max_	0.931, 1.000
No. of measured, independent and observed [*I* > 2σ(*I*)] reflections	13202, 7530, 3848
*R*_int_	0.062
(sin θ/λ)_max_ (Å^−1^)	0.629

Refinement	
*R*[*F*^2^ > 2σ(*F*^2^)], *wR*(*F*^2^), *S*	0.072, 0.192, 0.99
No. of reflections	7530
No. of parameters	578
H-atom treatment	H-atom parameters constrained
Δρ_max_, Δρ_min_ (e Å^−3^)	0.37, −0.35

Computer programs: *CrysAlis PRO* (Agilent, 2014 ▸), *SHELXT* (Sheldrick, 2015*a* ▸), *SHELXL2025/1* (Sheldrick, 2015*b* ▸) and *CrystalExplorer21.5* (Spackman *et al.*, 2021 ▸).

## supplementary crystallographic information

### Bis(µ-2-carboxy-4-nitrobenzoato-κ2O1:O1')bis[bis(1,10-phenanthroline-κ2N,N')cobalt(II)] bis(2-carboxy-4-nitrobenzoate) tetrahydrate Crystal data

**Table:**

[Co_2_(C_8_H_4_NO_6_)_2_(C_12_H_8_N_2_)_4_](C_8_H_4_NO_6_)_2_·4H_2_O	*Z* = 1
*M**_r_* = 1743.16	*F*(000) = 890
Triclinic, *P*1	*D*_x_ = 1.551 Mg m^−^^3^
*a* = 12.0908 (9) Å	Cu *K*α radiation, λ = 1.54184 Å
*b* = 13.2379 (15) Å	Cell parameters from 1481 reflections
*c* = 14.0783 (14) Å	θ = 3.5–76.0°
α = 111.315 (10)°	µ = 4.32 mm^−^^1^
β = 92.136 (7)°	*T* = 273 K
γ = 114.312 (9)°	Prism, red
*V* = 1866.1 (4) Å^3^	0.2 × 0.1 × 0.05 mm

### Bis(µ-2-carboxy-4-nitrobenzoato-κ2O1:O1')bis[bis(1,10-phenanthroline-κ2N,N')cobalt(II)] bis(2-carboxy-4-nitrobenzoate) tetrahydrate Data collection

**Table:**

XCalibur diffractometer	*R*_int_ = 0.062
ω scans	θ_max_ = 76.0°, θ_min_ = 3.5°
Absorption correction: multi-scan (CrysAlisPro; Agilent, 2014)	*h* = −9→15
*T*_min_ = 0.931, *T*_max_ = 1.000	*k* = −16→14
13202 measured reflections	*l* = −16→17
7530 independent reflections	3 standard reflections every 100 reflections
3848 reflections with *I* > 2σ(*I*)	intensity decay: 2.6%

### Bis(µ-2-carboxy-4-nitrobenzoato-κ2O1:O1')bis[bis(1,10-phenanthroline-κ2N,N')cobalt(II)] bis(2-carboxy-4-nitrobenzoate) tetrahydrate Refinement

**Table:**

Refinement on *F*^2^	0 restraints
Least-squares matrix: full	Hydrogen site location: inferred from neighbouring sites
*R*[*F*^2^ > 2σ(*F*^2^)] = 0.072	H-atom parameters constrained
*wR*(*F*^2^) = 0.192	*w* = 1/[σ^2^(*F*_o_^2^) + (0.0571*P*)^2^] where *P* = (*F*_o_^2^ + 2*F*_c_^2^)/3
*S* = 0.99	(Δ/σ)_max_ < 0.001
7530 reflections	Δρ_max_ = 0.37 e Å^−^^3^
578 parameters	Δρ_min_ = −0.35 e Å^−^^3^

### Bis(µ-2-carboxy-4-nitrobenzoato-κ2O1:O1')bis[bis(1,10-phenanthroline-κ2N,N')cobalt(II)] bis(2-carboxy-4-nitrobenzoate) tetrahydrate Special details

**Table:**

Geometry. All esds (except the esd in the dihedral angle between two l.s. planes) are estimated using the full covariance matrix. The cell esds are taken into account individually in the estimation of esds in distances, angles and torsion angles; correlations between esds in cell parameters are only used when they are defined by crystal symmetry. An approximate (isotropic) treatment of cell esds is used for estimating esds involving l.s. planes.

### Bis(µ-2-carboxy-4-nitrobenzoato-κ2O1:O1')bis[bis(1,10-phenanthroline-κ2N,N')cobalt(II)] bis(2-carboxy-4-nitrobenzoate) tetrahydrate Fractional atomic coordinates and isotropic or equivalent isotropic displacement parameters (Å^2^)

**Table:**

	*x*	*y*	*z*	*U*_iso_*/*U*_eq_	Occ. (<1)
Co1	0.63780 (7)	0.45259 (8)	0.58202 (7)	0.0501 (2)	
O6	0.5074 (3)	0.5019 (3)	0.4000 (3)	0.0529 (8)	
O5	0.6897 (3)	0.5301 (3)	0.4768 (3)	0.0573 (9)	
O4	0.5934 (4)	0.4004 (4)	0.2137 (3)	0.0831 (13)	
N5	0.5321 (4)	0.2729 (4)	0.4639 (3)	0.0549 (11)	
N4	0.5800 (4)	0.3470 (4)	0.6715 (3)	0.0549 (11)	
N3	0.7613 (4)	0.6286 (4)	0.6985 (3)	0.0572 (11)	
N2	0.8139 (4)	0.4459 (4)	0.5925 (3)	0.0535 (10)	
N6	0.0849 (5)	0.6768 (5)	0.2091 (4)	0.0727 (14)	
O12	0.1791 (4)	0.3410 (4)	0.0291 (4)	0.0993 (16)	
O3	0.5422 (5)	0.4770 (5)	0.1104 (3)	0.1009 (16)	
H3	0.502126	0.404406	0.071380	0.151*	
C32	0.0050 (5)	0.5470 (5)	0.1446 (4)	0.0575 (13)	
C34	−0.0120 (5)	0.3480 (5)	0.0469 (4)	0.0541 (13)	
O11	0.0133 (5)	0.1661 (4)	−0.0429 (4)	0.121 (2)	
H11A	−0.062094	0.139988	−0.047965	0.182*	
C29	0.8381 (5)	0.3532 (6)	0.5451 (5)	0.0650 (15)	
H29	0.771860	0.277347	0.504139	0.078*	
C31	0.9116 (4)	0.5571 (5)	0.6515 (4)	0.0507 (12)	
O10	0.1954 (4)	0.7124 (4)	0.2352 (4)	0.1085 (18)	
C8	0.6257 (5)	0.5512 (5)	0.4216 (4)	0.0533 (12)	
C35	−0.1421 (5)	0.2998 (5)	0.0286 (4)	0.0580 (13)	
C33	0.0596 (5)	0.4727 (5)	0.1059 (4)	0.0587 (13)	
H33	0.145924	0.506035	0.119276	0.070*	
C30	0.8836 (4)	0.6554 (5)	0.7057 (4)	0.0543 (13)	
C20	0.5052 (5)	0.2269 (5)	0.6125 (5)	0.0603 (14)	
O8	−0.3482 (5)	0.1418 (5)	−0.0361 (5)	0.138 (2)	
C18	0.5107 (5)	0.2382 (5)	0.3611 (4)	0.0650 (15)	
H18	0.548094	0.295772	0.334311	0.078*	
C19	0.4786 (5)	0.1873 (5)	0.5010 (4)	0.0594 (13)	
C7	0.6005 (6)	0.4889 (7)	0.1984 (5)	0.0717 (17)	
O9	0.0352 (5)	0.7429 (4)	0.2350 (5)	0.130 (2)	
C23	0.9800 (5)	0.7720 (5)	0.7650 (5)	0.0673 (15)	
C26	1.0366 (5)	0.5777 (6)	0.6641 (5)	0.0653 (15)	
C37	−0.1213 (5)	0.5033 (6)	0.1252 (5)	0.0715 (17)	
H37	−0.157196	0.555220	0.150060	0.086*	
C36	−0.1936 (5)	0.3802 (5)	0.0679 (5)	0.0683 (16)	
H36	−0.279661	0.349082	0.054714	0.082*	
C39	0.0671 (6)	0.2821 (6)	0.0091 (5)	0.0730 (17)	
C9	0.6056 (5)	0.3854 (6)	0.7739 (5)	0.0685 (16)	
H9	0.656358	0.467758	0.815176	0.082*	
O7	−0.2078 (5)	0.0856 (4)	−0.0594 (5)	0.134 (2)	
C25	1.1324 (5)	0.6983 (7)	0.7233 (5)	0.0771 (18)	
H25	1.214947	0.712587	0.729719	0.093*	
C10	0.5582 (6)	0.3052 (7)	0.8228 (5)	0.0783 (19)	
H10	0.578271	0.334874	0.895220	0.094*	
C40	0.7333 (5)	0.7166 (5)	0.7505 (5)	0.0701 (17)	
H40	0.649847	0.698499	0.746163	0.084*	
C28	0.9600 (6)	0.3637 (7)	0.5538 (5)	0.0770 (18)	
H28	0.973335	0.296045	0.521427	0.092*	
C38	−0.2404 (7)	0.1660 (6)	−0.0278 (5)	0.0808 (19)	
C17	0.4331 (6)	0.1173 (6)	0.2923 (5)	0.0811 (19)	
H17	0.421146	0.095545	0.220702	0.097*	
C27	1.0572 (6)	0.4764 (7)	0.6112 (5)	0.0772 (19)	
H27	1.138242	0.486258	0.615381	0.093*	
C15	0.3986 (6)	0.0643 (5)	0.4358 (6)	0.0768 (18)	
C24	1.1069 (5)	0.7926 (7)	0.7706 (5)	0.085 (2)	
H24	1.171526	0.870970	0.806768	0.102*	
C12	0.4542 (6)	0.1423 (6)	0.6554 (6)	0.0753 (17)	
C16	0.3757 (6)	0.0321 (6)	0.3275 (5)	0.086 (2)	
H16	0.321369	−0.047351	0.281117	0.104*	
C11	0.4843 (6)	0.1861 (7)	0.7649 (6)	0.0806 (19)	
H11	0.453254	0.132920	0.796878	0.097*	
C22	0.9462 (6)	0.8645 (6)	0.8177 (5)	0.086 (2)	
H22	1.007077	0.944291	0.856043	0.104*	
C21	0.8231 (6)	0.8357 (6)	0.8119 (6)	0.085 (2)	
H21	0.799544	0.895001	0.848520	0.102*	
C14	0.3459 (7)	−0.0191 (6)	0.4830 (6)	0.098 (2)	
H14	0.291867	−0.100385	0.440788	0.117*	
C13	0.3730 (7)	0.0181 (6)	0.5869 (6)	0.089 (2)	
H13	0.338160	−0.038425	0.615184	0.106*	
C1	0.6950 (5)	0.6438 (5)	0.3808 (5)	0.0634 (15)	
C2	0.7699 (6)	0.7631 (6)	0.4538 (7)	0.093 (2)	
H2	0.782074	0.780983	0.524832	0.112*	
C4	0.8068 (7)	0.8263 (8)	0.3159 (9)	0.105 (3)	
H4	0.844534	0.888375	0.293977	0.126*	0.402 (7)
C5	0.7353 (6)	0.7117 (7)	0.2422 (6)	0.086 (2)	
H5	0.725177	0.695380	0.171541	0.103*	
C6	0.6763 (5)	0.6172 (6)	0.2756 (5)	0.0659 (15)	
N1	0.8655 (10)	0.9355 (10)	0.3002 (11)	0.105 (4)	0.598 (7)
O1	0.947 (2)	1.0306 (18)	0.3634 (16)	0.228 (13)	0.598 (7)
O2	0.8274 (10)	0.9203 (8)	0.2134 (8)	0.119 (4)	0.598 (7)
O1A	0.8979 (19)	1.0175 (17)	0.5584 (17)	0.154 (9)	0.402 (7)
N1A	0.8839 (17)	0.9840 (18)	0.467 (2)	0.125 (9)	0.402 (7)
O2A	0.919 (2)	1.0523 (18)	0.414 (2)	0.137 (12)	0.402 (7)
C3	0.8262 (8)	0.8554 (8)	0.4204 (10)	0.122 (3)	
H3A	0.876183	0.935342	0.468435	0.147*	0.598 (7)
O1W	0.3637 (5)	0.2669 (5)	−0.0019 (3)	0.1107 (18)	
O2W	0.3993 (5)	0.0657 (6)	0.9609 (4)	0.153 (3)	

### Bis(µ-2-carboxy-4-nitrobenzoato-κ2O1:O1')bis[bis(1,10-phenanthroline-κ2N,N')cobalt(II)] bis(2-carboxy-4-nitrobenzoate) tetrahydrate Atomic displacement parameters (Å^2^)

**Table:**

	*U*^11^	*U*^22^	*U*^33^	*U*^12^	*U*^13^	*U*^23^
Co1	0.0440 (4)	0.0457 (4)	0.0549 (5)	0.0204 (3)	0.0095 (3)	0.0152 (4)
O6	0.0457 (19)	0.053 (2)	0.060 (2)	0.0243 (16)	0.0158 (16)	0.0210 (17)
O5	0.0468 (19)	0.070 (2)	0.068 (2)	0.0323 (18)	0.0182 (17)	0.035 (2)
O4	0.109 (4)	0.083 (3)	0.075 (3)	0.054 (3)	0.036 (3)	0.037 (3)
N5	0.054 (2)	0.048 (2)	0.060 (3)	0.024 (2)	0.010 (2)	0.019 (2)
N4	0.048 (2)	0.062 (3)	0.063 (3)	0.028 (2)	0.018 (2)	0.029 (2)
N3	0.057 (3)	0.050 (2)	0.059 (3)	0.026 (2)	0.007 (2)	0.017 (2)
N2	0.059 (3)	0.053 (3)	0.058 (3)	0.031 (2)	0.016 (2)	0.025 (2)
N6	0.083 (4)	0.055 (3)	0.075 (3)	0.031 (3)	0.019 (3)	0.021 (3)
O12	0.067 (3)	0.085 (3)	0.132 (4)	0.040 (3)	0.020 (3)	0.024 (3)
O3	0.136 (4)	0.116 (4)	0.069 (3)	0.075 (4)	0.022 (3)	0.037 (3)
C32	0.063 (3)	0.047 (3)	0.056 (3)	0.021 (3)	0.014 (3)	0.019 (3)
C34	0.060 (3)	0.051 (3)	0.053 (3)	0.027 (3)	0.013 (2)	0.022 (3)
O11	0.110 (4)	0.074 (3)	0.145 (5)	0.055 (3)	0.004 (4)	−0.005 (3)
C29	0.067 (4)	0.072 (4)	0.073 (4)	0.042 (3)	0.025 (3)	0.035 (3)
C31	0.044 (3)	0.063 (3)	0.055 (3)	0.027 (2)	0.013 (2)	0.032 (3)
O10	0.074 (3)	0.063 (3)	0.132 (5)	0.017 (2)	0.002 (3)	0.000 (3)
C8	0.050 (3)	0.050 (3)	0.056 (3)	0.024 (2)	0.017 (2)	0.016 (2)
C35	0.059 (3)	0.049 (3)	0.055 (3)	0.016 (3)	0.017 (3)	0.020 (3)
C33	0.062 (3)	0.055 (3)	0.058 (3)	0.025 (3)	0.011 (3)	0.024 (3)
C30	0.044 (3)	0.058 (3)	0.058 (3)	0.020 (2)	0.009 (2)	0.025 (3)
C20	0.054 (3)	0.048 (3)	0.079 (4)	0.025 (3)	0.016 (3)	0.026 (3)
O8	0.069 (3)	0.081 (4)	0.193 (6)	0.004 (3)	0.037 (4)	0.017 (4)
C18	0.078 (4)	0.058 (3)	0.057 (3)	0.034 (3)	0.009 (3)	0.020 (3)
C19	0.062 (3)	0.049 (3)	0.062 (3)	0.024 (3)	0.011 (3)	0.019 (3)
C7	0.083 (4)	0.101 (5)	0.062 (4)	0.059 (4)	0.039 (3)	0.044 (4)
O9	0.114 (4)	0.061 (3)	0.181 (6)	0.050 (3)	0.018 (4)	0.004 (3)
C23	0.057 (3)	0.062 (4)	0.075 (4)	0.020 (3)	0.006 (3)	0.030 (3)
C26	0.054 (3)	0.093 (5)	0.062 (3)	0.035 (3)	0.023 (3)	0.042 (3)
C37	0.068 (4)	0.066 (4)	0.084 (4)	0.037 (3)	0.027 (3)	0.027 (3)
C36	0.052 (3)	0.066 (4)	0.086 (4)	0.026 (3)	0.022 (3)	0.032 (3)
C39	0.084 (5)	0.064 (4)	0.070 (4)	0.041 (4)	0.010 (3)	0.019 (3)
C9	0.057 (3)	0.082 (4)	0.064 (4)	0.037 (3)	0.011 (3)	0.021 (3)
O7	0.101 (4)	0.053 (3)	0.181 (6)	0.026 (3)	−0.002 (4)	−0.006 (3)
C25	0.043 (3)	0.103 (5)	0.086 (5)	0.025 (3)	0.012 (3)	0.050 (4)
C10	0.081 (4)	0.118 (6)	0.064 (4)	0.057 (4)	0.026 (3)	0.052 (4)
C40	0.061 (3)	0.047 (3)	0.082 (4)	0.026 (3)	0.005 (3)	0.006 (3)
C28	0.097 (5)	0.099 (5)	0.078 (4)	0.071 (4)	0.038 (4)	0.048 (4)
C38	0.087 (5)	0.061 (4)	0.080 (5)	0.026 (4)	0.020 (4)	0.024 (3)
C17	0.099 (5)	0.059 (4)	0.064 (4)	0.033 (4)	−0.002 (4)	0.008 (3)
C27	0.074 (4)	0.122 (6)	0.083 (5)	0.067 (4)	0.040 (4)	0.063 (5)
C15	0.077 (4)	0.049 (3)	0.093 (5)	0.022 (3)	0.012 (4)	0.025 (3)
C24	0.051 (3)	0.084 (5)	0.090 (5)	0.004 (3)	0.001 (3)	0.037 (4)
C12	0.070 (4)	0.073 (4)	0.092 (5)	0.032 (3)	0.024 (4)	0.043 (4)
C16	0.094 (5)	0.049 (4)	0.080 (5)	0.021 (3)	−0.009 (4)	0.006 (3)
C11	0.083 (5)	0.088 (5)	0.088 (5)	0.044 (4)	0.023 (4)	0.048 (4)
C22	0.080 (4)	0.053 (4)	0.085 (5)	0.011 (3)	−0.001 (4)	0.009 (3)
C21	0.081 (4)	0.052 (4)	0.102 (5)	0.031 (3)	0.003 (4)	0.011 (4)
C14	0.106 (6)	0.045 (4)	0.116 (7)	0.020 (4)	0.016 (5)	0.023 (4)
C13	0.101 (5)	0.063 (4)	0.103 (6)	0.027 (4)	0.032 (4)	0.046 (4)
C1	0.048 (3)	0.068 (4)	0.085 (4)	0.031 (3)	0.017 (3)	0.038 (3)
C2	0.065 (4)	0.063 (4)	0.122 (6)	0.012 (3)	0.013 (4)	0.028 (4)
C4	0.067 (5)	0.085 (6)	0.179 (9)	0.025 (4)	0.035 (6)	0.081 (7)
C5	0.070 (4)	0.100 (6)	0.115 (6)	0.042 (4)	0.037 (4)	0.067 (5)
C6	0.056 (3)	0.070 (4)	0.092 (5)	0.037 (3)	0.030 (3)	0.044 (4)
N1	0.086 (7)	0.081 (8)	0.123 (10)	−0.004 (6)	−0.005 (7)	0.068 (8)
O1	0.23 (2)	0.153 (15)	0.138 (14)	−0.074 (13)	−0.068 (14)	0.089 (12)
O2	0.141 (9)	0.092 (7)	0.120 (8)	0.025 (6)	0.020 (6)	0.072 (6)
O1A	0.141 (16)	0.096 (13)	0.19 (2)	0.059 (11)	0.008 (16)	0.012 (14)
N1A	0.068 (10)	0.073 (13)	0.20 (3)	0.021 (9)	0.017 (15)	0.038 (16)
O2A	0.087 (10)	0.063 (10)	0.28 (4)	0.027 (9)	0.078 (17)	0.101 (17)
C3	0.092 (6)	0.074 (6)	0.190 (11)	0.026 (5)	0.031 (7)	0.059 (7)
O1W	0.107 (4)	0.131 (5)	0.097 (4)	0.071 (4)	0.023 (3)	0.030 (3)
O2W	0.098 (4)	0.147 (6)	0.254 (8)	0.048 (4)	0.053 (5)	0.132 (6)

### Bis(µ-2-carboxy-4-nitrobenzoato-κ2O1:O1')bis[bis(1,10-phenanthroline-κ2N,N')cobalt(II)] bis(2-carboxy-4-nitrobenzoate) tetrahydrate Geometric parameters (Å, º)

**Table:**

Co1—O5	2.070 (4)	C26—C27	1.406 (9)
Co1—O6^i^	2.073 (3)	C26—C25	1.422 (9)
Co1—N5	2.126 (4)	C37—C36	1.374 (8)
Co1—N3	2.126 (4)	C37—H37	0.9300
Co1—N4	2.137 (5)	C36—H36	0.9300
Co1—N2	2.169 (4)	C9—C10	1.410 (8)
O6—C8	1.273 (6)	C9—H9	0.9300
O5—C8	1.254 (6)	O7—C38	1.225 (8)
O4—C7	1.237 (7)	C25—C24	1.351 (9)
N5—C18	1.328 (7)	C25—H25	0.9300
N5—C19	1.351 (7)	C10—C11	1.342 (9)
N4—C9	1.319 (7)	C10—H10	0.9300
N4—C20	1.355 (7)	C40—C21	1.393 (8)
N3—C40	1.316 (6)	C40—H40	0.9300
N3—C30	1.361 (6)	C28—C27	1.368 (9)
N2—C29	1.324 (7)	C28—H28	0.9300
N2—C31	1.363 (6)	C17—C16	1.336 (9)
N6—O10	1.210 (6)	C17—H17	0.9300
N6—O9	1.215 (6)	C27—H27	0.9300
N6—C32	1.464 (7)	C15—C16	1.407 (9)
O12—C39	1.209 (7)	C15—C14	1.432 (9)
O3—C7	1.326 (7)	C24—H24	0.9300
O3—H3	0.8200	C12—C11	1.407 (9)
C32—C33	1.367 (7)	C12—C13	1.430 (9)
C32—C37	1.367 (7)	C16—H16	0.9300
C34—C33	1.392 (7)	C11—H11	0.9300
C34—C35	1.404 (7)	C22—C21	1.366 (9)
C34—C39	1.532 (7)	C22—H22	0.9300
O11—C39	1.281 (7)	C21—H21	0.9300
O11—H11A	0.8200	C14—C13	1.342 (9)
C29—C28	1.418 (8)	C14—H14	0.9300
C29—H29	0.9300	C13—H13	0.9300
C31—C26	1.412 (7)	C1—C6	1.378 (8)
C31—C30	1.432 (7)	C1—C2	1.394 (9)
C8—C1	1.496 (8)	C2—C3	1.391 (11)
C35—C36	1.403 (7)	C2—H2	0.9300
C35—C38	1.534 (8)	C4—C5	1.354 (11)
C33—H33	0.9300	C4—C3	1.363 (13)
C30—C23	1.395 (8)	C4—N1	1.428 (12)
C20—C12	1.398 (8)	C4—H4	0.9300
C20—C19	1.437 (8)	C5—C6	1.418 (8)
O8—C38	1.197 (8)	C5—H5	0.9300
C18—C17	1.396 (8)	N1—O2	1.204 (13)
C18—H18	0.9300	N1—O1	1.20 (2)
C19—C15	1.407 (8)	O1A—N1A	1.18 (3)
C7—C6	1.485 (9)	N1A—O2A	1.32 (3)
C23—C22	1.411 (9)	N1A—C3	1.41 (2)
C23—C24	1.437 (8)	C3—H3A	0.9300
			
O5—Co1—O6^i^	91.94 (13)	O12—C39—O11	121.6 (6)
O5—Co1—N5	94.13 (16)	O12—C39—C34	119.1 (6)
O6^i^—Co1—N5	96.86 (15)	O11—C39—C34	119.3 (6)
O5—Co1—N3	85.40 (17)	N4—C9—C10	121.9 (6)
O6^i^—Co1—N3	89.61 (15)	N4—C9—H9	119.0
N5—Co1—N3	173.52 (16)	C10—C9—H9	119.0
O5—Co1—N4	171.16 (16)	C24—C25—C26	121.8 (6)
O6^i^—Co1—N4	92.48 (15)	C24—C25—H25	119.1
N5—Co1—N4	77.74 (18)	C26—C25—H25	119.1
N3—Co1—N4	102.28 (17)	C11—C10—C9	120.1 (6)
O5—Co1—N2	87.92 (15)	C11—C10—H10	120.0
O6^i^—Co1—N2	166.84 (15)	C9—C10—H10	120.0
N5—Co1—N2	96.27 (16)	N3—C40—C21	122.9 (6)
N3—Co1—N2	77.25 (16)	N3—C40—H40	118.5
N4—Co1—N2	89.51 (16)	C21—C40—H40	118.5
C8—O6—Co1^i^	140.8 (3)	C27—C28—C29	118.0 (6)
C8—O5—Co1	128.7 (3)	C27—C28—H28	121.0
C18—N5—C19	118.3 (5)	C29—C28—H28	121.0
C18—N5—Co1	127.5 (4)	O8—C38—O7	121.0 (7)
C19—N5—Co1	114.1 (4)	O8—C38—C35	119.0 (6)
C9—N4—C20	118.3 (5)	O7—C38—C35	119.9 (6)
C9—N4—Co1	128.1 (4)	C16—C17—C18	121.2 (7)
C20—N4—Co1	113.6 (4)	C16—C17—H17	119.4
C40—N3—C30	118.3 (5)	C18—C17—H17	119.4
C40—N3—Co1	127.0 (4)	C28—C27—C26	121.0 (6)
C30—N3—Co1	113.9 (3)	C28—C27—H27	119.5
C29—N2—C31	118.0 (5)	C26—C27—H27	119.5
C29—N2—Co1	129.2 (4)	C19—C15—C16	116.9 (6)
C31—N2—Co1	112.6 (3)	C19—C15—C14	118.6 (6)
O10—N6—O9	123.5 (6)	C16—C15—C14	124.5 (6)
O10—N6—C32	118.9 (5)	C25—C24—C23	120.1 (6)
O9—N6—C32	117.6 (5)	C25—C24—H24	120.0
C7—O3—H3	109.5	C23—C24—H24	120.0
C33—C32—C37	121.9 (5)	C20—C12—C11	117.3 (6)
C33—C32—N6	118.7 (5)	C20—C12—C13	118.8 (6)
C37—C32—N6	119.4 (5)	C11—C12—C13	123.9 (7)
C33—C34—C35	118.3 (5)	C17—C16—C15	119.1 (6)
C33—C34—C39	112.7 (5)	C17—C16—H16	120.4
C35—C34—C39	129.0 (5)	C15—C16—H16	120.4
C39—O11—H11A	109.5	C10—C11—C12	119.5 (7)
N2—C29—C28	123.3 (6)	C10—C11—H11	120.3
N2—C29—H29	118.4	C12—C11—H11	120.3
C28—C29—H29	118.4	C21—C22—C23	119.4 (6)
N2—C31—C26	123.2 (5)	C21—C22—H22	120.3
N2—C31—C30	117.3 (4)	C23—C22—H22	120.3
C26—C31—C30	119.4 (5)	C22—C21—C40	119.4 (6)
O5—C8—O6	124.9 (5)	C22—C21—H21	120.3
O5—C8—C1	116.9 (5)	C40—C21—H21	120.3
O6—C8—C1	118.2 (5)	C13—C14—C15	121.1 (7)
C36—C35—C34	118.4 (5)	C13—C14—H14	119.5
C36—C35—C38	113.2 (5)	C15—C14—H14	119.5
C34—C35—C38	128.3 (5)	C14—C13—C12	121.7 (7)
C32—C33—C34	121.0 (5)	C14—C13—H13	119.1
C32—C33—H33	119.5	C12—C13—H13	119.1
C34—C33—H33	119.5	C6—C1—C2	120.2 (7)
N3—C30—C23	122.9 (5)	C6—C1—C8	122.0 (6)
N3—C30—C31	117.2 (5)	C2—C1—C8	117.4 (6)
C23—C30—C31	119.9 (5)	C3—C2—C1	119.9 (9)
N4—C20—C12	122.9 (6)	C3—C2—H2	120.0
N4—C20—C19	117.3 (5)	C1—C2—H2	120.0
C12—C20—C19	119.8 (6)	C5—C4—C3	123.4 (9)
N5—C18—C17	121.4 (6)	C5—C4—N1	127.5 (11)
N5—C18—H18	119.3	C3—C4—N1	109.0 (10)
C17—C18—H18	119.3	C5—C4—H4	118.3
N5—C19—C15	122.9 (6)	C3—C4—H4	118.3
N5—C19—C20	117.1 (5)	C4—C5—C6	118.4 (8)
C15—C19—C20	120.0 (6)	C4—C5—H5	120.8
O4—C7—O3	122.5 (7)	C6—C5—H5	120.8
O4—C7—C6	123.2 (6)	C1—C6—C5	119.4 (7)
O3—C7—C6	114.3 (6)	C1—C6—C7	119.6 (6)
C30—C23—C22	117.1 (5)	C5—C6—C7	120.9 (7)
C30—C23—C24	119.6 (6)	O2—N1—O1	123.0 (14)
C22—C23—C24	123.2 (6)	O2—N1—C4	111.8 (11)
C27—C26—C31	116.5 (6)	O1—N1—C4	125.0 (13)
C27—C26—C25	124.5 (6)	O1A—N1A—O2A	127 (2)
C31—C26—C25	118.9 (6)	O1A—N1A—C3	108 (3)
C32—C37—C36	117.8 (5)	O2A—N1A—C3	124 (2)
C32—C37—H37	121.1	C4—C3—C2	118.7 (9)
C36—C37—H37	121.1	C4—C3—N1A	103.8 (15)
C37—C36—C35	122.4 (5)	C2—C3—N1A	136.2 (16)
C37—C36—H36	118.8	C4—C3—H3A	120.7
C35—C36—H36	118.8	C2—C3—H3A	120.7
			
O10—N6—C32—C33	−7.4 (9)	Co1—N3—C40—C21	−168.4 (5)
O9—N6—C32—C33	173.6 (6)	N2—C29—C28—C27	−2.3 (9)
O10—N6—C32—C37	172.2 (6)	C36—C35—C38—O8	1.9 (10)
O9—N6—C32—C37	−6.8 (9)	C34—C35—C38—O8	179.9 (7)
C31—N2—C29—C28	0.7 (8)	C36—C35—C38—O7	−174.8 (7)
Co1—N2—C29—C28	175.7 (4)	C34—C35—C38—O7	3.3 (11)
C29—N2—C31—C26	0.2 (8)	N5—C18—C17—C16	1.2 (10)
Co1—N2—C31—C26	−175.6 (4)	C29—C28—C27—C26	2.9 (9)
C29—N2—C31—C30	−177.5 (5)	C31—C26—C27—C28	−2.1 (9)
Co1—N2—C31—C30	6.7 (6)	C25—C26—C27—C28	−178.5 (6)
Co1—O5—C8—O6	−18.6 (7)	N5—C19—C15—C16	0.1 (9)
Co1—O5—C8—C1	159.8 (4)	C20—C19—C15—C16	179.4 (6)
Co1^i^—O6—C8—O5	119.9 (5)	N5—C19—C15—C14	−179.6 (6)
Co1^i^—O6—C8—C1	−58.5 (8)	C20—C19—C15—C14	−0.3 (9)
C33—C34—C35—C36	1.9 (8)	C26—C25—C24—C23	2.3 (10)
C39—C34—C35—C36	−177.0 (6)	C30—C23—C24—C25	−3.6 (10)
C33—C34—C35—C38	−176.1 (6)	C22—C23—C24—C25	175.9 (7)
C39—C34—C35—C38	5.0 (10)	N4—C20—C12—C11	−0.6 (9)
C37—C32—C33—C34	−1.6 (9)	C19—C20—C12—C11	179.5 (5)
N6—C32—C33—C34	178.0 (5)	N4—C20—C12—C13	178.0 (6)
C35—C34—C33—C32	−0.5 (8)	C19—C20—C12—C13	−1.9 (9)
C39—C34—C33—C32	178.6 (5)	C18—C17—C16—C15	−2.9 (11)
C40—N3—C30—C23	−0.5 (9)	C19—C15—C16—C17	2.2 (10)
Co1—N3—C30—C23	169.7 (5)	C14—C15—C16—C17	−178.1 (7)
C40—N3—C30—C31	177.7 (5)	C9—C10—C11—C12	−0.3 (10)
Co1—N3—C30—C31	−12.1 (6)	C20—C12—C11—C10	0.7 (10)
N2—C31—C30—N3	3.5 (7)	C13—C12—C11—C10	−177.8 (7)
C26—C31—C30—N3	−174.3 (5)	C30—C23—C22—C21	2.5 (10)
N2—C31—C30—C23	−178.3 (5)	C24—C23—C22—C21	−177.1 (7)
C26—C31—C30—C23	3.9 (8)	C23—C22—C21—C40	−2.6 (11)
C9—N4—C20—C12	0.1 (8)	N3—C40—C21—C22	1.2 (12)
Co1—N4—C20—C12	−178.2 (4)	C19—C15—C14—C13	−1.0 (11)
C9—N4—C20—C19	179.9 (5)	C16—C15—C14—C13	179.3 (7)
Co1—N4—C20—C19	1.7 (6)	C15—C14—C13—C12	0.8 (12)
C19—N5—C18—C17	1.2 (8)	C20—C12—C13—C14	0.7 (11)
Co1—N5—C18—C17	−176.2 (4)	C11—C12—C13—C14	179.2 (7)
C18—N5—C19—C15	−1.8 (8)	O5—C8—C1—C6	123.9 (5)
Co1—N5—C19—C15	175.9 (5)	O6—C8—C1—C6	−57.6 (7)
C18—N5—C19—C20	178.9 (5)	O5—C8—C1—C2	−63.1 (7)
Co1—N5—C19—C20	−3.4 (6)	O6—C8—C1—C2	115.3 (6)
N4—C20—C19—N5	1.2 (7)	C6—C1—C2—C3	−0.3 (10)
C12—C20—C19—N5	−179.0 (5)	C8—C1—C2—C3	−173.4 (6)
N4—C20—C19—C15	−178.2 (5)	C3—C4—C5—C6	0.8 (13)
C12—C20—C19—C15	1.7 (8)	N1—C4—C5—C6	−174.3 (9)
N3—C30—C23—C22	−0.9 (9)	C2—C1—C6—C5	1.0 (8)
C31—C30—C23—C22	−179.1 (5)	C8—C1—C6—C5	173.8 (5)
N3—C30—C23—C24	178.6 (5)	C2—C1—C6—C7	177.5 (5)
C31—C30—C23—C24	0.5 (9)	C8—C1—C6—C7	−9.7 (8)
N2—C31—C26—C27	0.5 (8)	C4—C5—C6—C1	−1.2 (9)
C30—C31—C26—C27	178.1 (5)	C4—C5—C6—C7	−177.8 (6)
N2—C31—C26—C25	177.1 (5)	O4—C7—C6—C1	−29.9 (9)
C30—C31—C26—C25	−5.2 (8)	O3—C7—C6—C1	151.2 (5)
C33—C32—C37—C36	2.1 (9)	O4—C7—C6—C5	146.6 (6)
N6—C32—C37—C36	−177.4 (6)	O3—C7—C6—C5	−32.3 (8)
C32—C37—C36—C35	−0.6 (10)	C5—C4—N1—O2	12.5 (18)
C34—C35—C36—C37	−1.4 (9)	C3—C4—N1—O2	−163.2 (12)
C38—C35—C36—C37	176.9 (6)	C5—C4—N1—O1	−164.2 (19)
C33—C34—C39—O12	−2.5 (9)	C3—C4—N1—O1	20 (2)
C35—C34—C39—O12	176.5 (6)	C5—C4—C3—C2	−0.1 (14)
C33—C34—C39—O11	177.4 (6)	N1—C4—C3—C2	175.8 (8)
C35—C34—C39—O11	−3.6 (10)	C5—C4—C3—N1A	−169.0 (11)
C20—N4—C9—C10	0.4 (8)	C1—C2—C3—C4	−0.2 (13)
Co1—N4—C9—C10	178.4 (4)	C1—C2—C3—N1A	164.1 (15)
C27—C26—C25—C24	178.5 (6)	O1A—N1A—C3—C4	176.0 (18)
C31—C26—C25—C24	2.2 (10)	O2A—N1A—C3—C4	−6 (2)
N4—C9—C10—C11	−0.3 (9)	O1A—N1A—C3—C2	10 (3)
C30—N3—C40—C21	0.4 (10)	O2A—N1A—C3—C2	−172.0 (15)

Symmetry code: (i) −*x*+1, −*y*+1, −*z*+1.

### Bis(µ-2-carboxy-4-nitrobenzoato-κ2O1:O1')bis[bis(1,10-phenanthroline-κ2N,N')cobalt(II)] bis(2-carboxy-4-nitrobenzoate) tetrahydrate Hydrogen-bond geometry (Å, º)

**Table:**

*D*—H···*A*	*D*—H	H···*A*	*D*···*A*	*D*—H···*A*
O3—H3···O1*W*	0.82	1.78	2.549 (8)	155
O11—H11*A*···O7	0.82	1.58	2.400 (8)	178
C18—H18···O4	0.93	2.50	3.397 (8)	162
C9—H9···O12^i^	0.93	2.58	3.462 (8)	158
C40—H40···O6^i^	0.93	2.54	3.079 (6)	117
C21—H21···O7^ii^	0.93	2.42	3.329 (8)	165
C4—H4^b···O2^*^A^*b	0.93	2.00	2.49 (2)	112

Symmetry codes: (i) −*x*+1, −*y*+1, −*z*+1; (ii) *x*+1, *y*+1, *z*+1.
